# Quasi-Static Calibration Method of a High-g Accelerometer

**DOI:** 10.3390/s17020409

**Published:** 2017-02-20

**Authors:** Yan Wang, Jinbiao Fan, Jing Zu, Peng Xu

**Affiliations:** 1 Science and Technology on Electronic Test and Measurement Laboratory, North University of China, Taiyuan 030051, China; jingzu@263.net (J.Z.); ncitlxpx@nuc.edu.cn (P.X.); 2 School of Computer Science and Control Engineering, North University of China, Taiyuan 030051, China; fanjinbiao@nuc.edu.cn

**Keywords:** quasi-static calibration, high-g accelerometer, pulse width, gas gun, laser interferometer

## Abstract

To solve the problem of resonance during quasi-static calibration of high-g accelerometers, we deduce the relationship between the minimum excitation pulse width and the resonant frequency of the calibrated accelerometer according to the second-order mathematical model of the accelerometer, and improve the quasi-static calibration theory. We establish a quasi-static calibration testing system, which uses a gas gun to generate high-g acceleration signals, and apply a laser interferometer to reproduce the impact acceleration. These signals are used to drive the calibrated accelerometer. By comparing the excitation acceleration signal and the output responses of the calibrated accelerometer to the excitation signals, the impact sensitivity of the calibrated accelerometer is obtained. As indicated by the calibration test results, this calibration system produces excitation acceleration signals with a pulse width of less than 1000 μs, and realize the quasi-static calibration of high-g accelerometers with a resonant frequency above 20 kHz when the calibration error was 3%.

## 1. Introduction

High-g accelerometers are designed to measure impact acceleration when launching a projectile body from the barrel of a gun, penetrating a target, etc. The impact sensitivity and uncertainty of the calibrated accelerometer are obtained through the quasi-static calibration test to guarantee accuracy of the high-impact test data. This is of great significance to both theoretical research on weapon design, and analyses on weapon performance [1,2,3,4,5].

Physikalisch Technische Bundesanstalt (PTB) takes the lead in establishing a high-impact calibration system and uses laser interferometry to achieve the absolute calibration of the accelerometer. An acceleration peak of 10^2^–10^4^ g and an impact pulse width of 30–300 μs can be produced, with an expanded uncertainty of the measured impact sensitivity results of 1% [6,7]. Additionally, Wang apply a differential-type laser Doppler velocity-measuring system to conduct an impact calibration of an accelerometer, with a measuring range of 10^5^ g, and measurement uncertainty is less than 5% [8]. The National Institute of Metrology (NRLM) conduct a series of studies on the dynamic characteristics of accelerometers in the range of the reference acceleration peak of 20–10^4^ g and the impulse duration of 10–100 μs [9].

Many metrology institutes refer to the ISO16063-13 international standard [10] and establish a standard impact calibration system [11,12,13] composed by the impact excitation system and laser interferometer. Within the range of 10^5^ g (the peak value of impact acceleration) and 200 μs (the pulse duration), the absolute calibration of the impact sensitivity of a high-g accelerometer is achieved with a calibration uncertainty of 5% [13,14]. In recent years, the measurement uncertainty caused by this measurement method has been studied [15,16].

Impact sensitivity is related to the features of excitation signals, such as peak value, pulse duration, etc. In the impact calibration test, the output signal of the calibrated accelerometer is obtained after digital filtering, and the filter cut-off frequency influences the signal peak. Especially when the signal contains certain resonant components, such an influence will become more obvious [10,12,13]. To avoid resonance of the high-g accelerometer, the quasi-static calibration method for high-g accelerometers should be improved.

By studying the relationship between the pulse width of the excitation signal and the resonant frequency of the high-g accelerometer, the present study improve the quasi-static calibration theory. We use a gas gun testing system to generate high-g half-sine acceleration signals with a wide pulse width, and drive the accelerometer with these signals. By comparing the responses of the excitation acceleration signals and the calibrated accelerometer to the excitation signals, we obtain the impact sensitivity of the calibrated accelerometer.

## 2. Principle of Quasi-Static Calibration

### 2.1. Principle of Quasi-Static Calibration of High-g Accelerometer

It is hard to realize the static calibration of high-g accelerometer, but it can usually be conducted in a quasi-static state. Quasi-static calibration excites the calibrated system using an extremely low amount of high-frequency of excitation signals, in the hope that the proportion occupied by the inherent frequency component of the calibrated system in its response output can be small and within a pre-specified range, namely, exciting the accelerometer with wide-pulse acceleration signals. Meanwhile, the excitation signals (obtained by standard instruments) that can be traced to the source function as the input signals, and the impact sensitivity and relative uncertainty of the accelerometer are calculated after multiple fittings. We implement this calibration method in this study.

**Figure 1:**
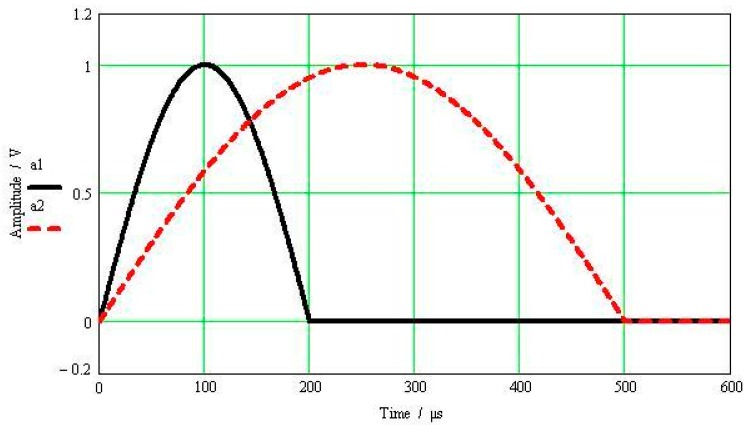
Half-sine excitation pulse.

**Figure 2:**
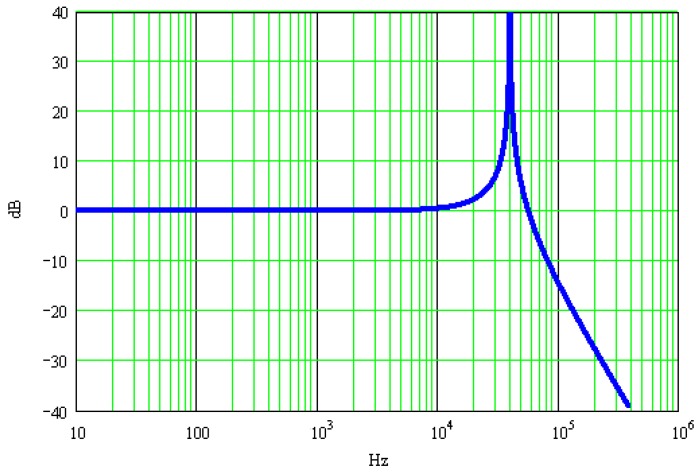
Amplitude-frequency characteristics of the accelerometer.

**Figure 3:**
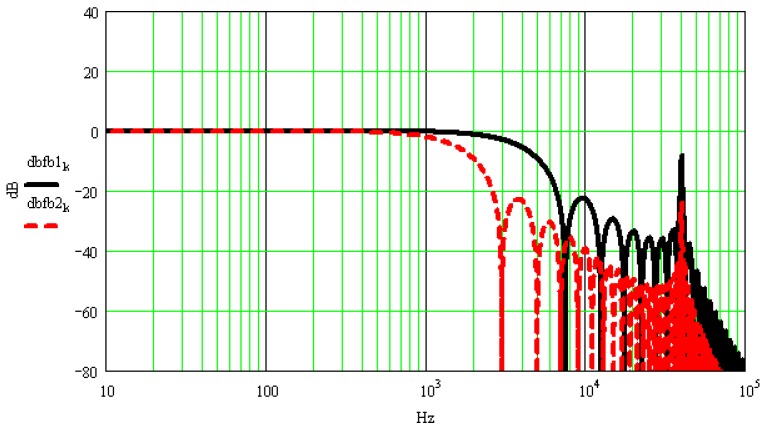
Sensor’s frequency domain response to the two pulsed excitations.

**Figure 4:**
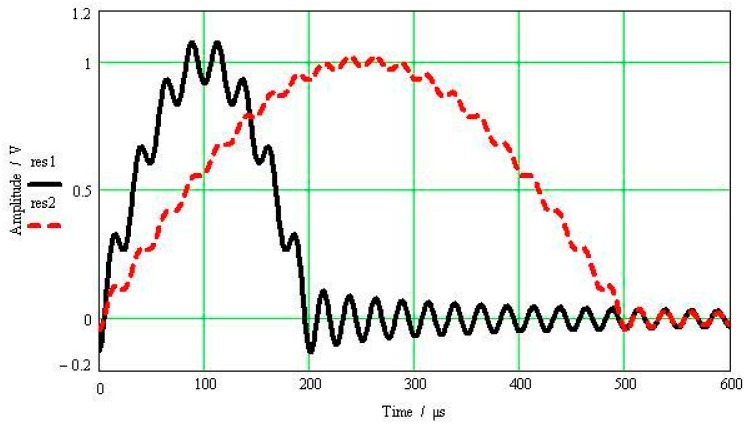
Sensor’s time domain response to the two pulsed excitations.

Figure 1 shows half-sine excitation signals with amplitude of 1 V and pulse widths being 500 μs and 200 μs, respectively. The resonant frequency of the calibrated accelerometer is 40 kHz, its resonance peak is 40 dB and its normalized amplitude-frequency characteristics are shown in Figure 2. Figure 3 shows the calibrated accelerometer’s frequency domain response to the two pulsed excitations, and Figure 4 demonstrates the calibrated accelerometer’s time domain response to the two pulse excitations gained by Fourier inversion.

As shown in Figure 4, the response and excitation signals change with the same trend, and the oscillation curve superimposed on the response signal is the response error. The narrower the pulse width of the excitation signal, the wider the frequency range covered by the main lobe width of the corresponding frequency spectrum, and thus the higher the modal frequency the accelerometer can excite. This then results in a larger amplitude error between the response and excitation signals. As shown in Figure 4, the amplitude error of the half-sine signal with a pulse width of 200 μs reaches 7.4%, while that of the half-sine signal with a pulse width of 500 μs reaches 1.9%.

Since there are a large variety of high-g accelerometers with different resonant frequencies, abundant tests were required to determine the pulse width of the excitation signal so as not to arouse the resonant frequency of the accelerometer, which increases the workload of the calibration test. Therefore, to achieve quasi-static calibration of high-g accelerometers, the minimum pulse width of excitation signals with different resonant frequencies must be theoretically determined within a specific response error [17].

### 2.2. Minimum Pulse Width of Excitation Signal Required for Quasi-Static Calibration

High-g accelerometers can be divided into two types, namely the piezoelectric type and the piezoresistive type. Although they have different principles and structures, both can be approximately equivalent to the spring-mass system, namely, the response characteristics of the accelerometer can be described by a second-order mathematical model.

The mathematical model of the accelerometer can be expressed as in Equation (1):

(1) $$m\ddot{y}+c\dot{y}+ky=-m\ddot{x}$$

where m denotes mass weight; c denotes the damping coefficient; k denotes the rigidity coefficient; y is the displacement of the mass relative to the base; $\ddot{x}$ denotes the excitation acceleration. Let $\alpha =\frac{\ddot{x}}{{\ddot{x}}_{m}}$, $\theta =\frac{t}{\tau }$, $\zeta =\frac{c_{c}}{c}$, $c_{c}=2\sqrt{km}$, $\delta =-\frac{ky}{m{\ddot{x}}_{m}}$, $R=\frac{T_{n}}{\tau }$, $T_{n}=2\pi \sqrt{m/k}=\frac{2\pi }{\omega_{n}}$, then Equation (1) can be rewritten as:

(2) $${\left(\frac{R}{2\pi }\right)}^{2}\frac{d^{2}\phi }{d\theta^{2}}+\left(\frac{R\zeta }{\pi }\right)\frac{d\phi }{d\theta }+\phi =\alpha$$

where α denotes the dimensionless excitation acceleration; ${\ddot{x}}_{m}$ is the peak value of the excitation acceleration; $\ddot{x}$ denotes the instantaneous value of the excitation acceleration; θ refers to the dimensionless time; τ denotes the pulse duration of the excitation acceleration; $c_{c}$ is the critical damping coefficient; ϕ is the dimensionless response acceleration, which is relative to the dimensionless input α; $T_{n}$ represents the undamped natural period of the accelerometer; and R stands for the ratio of the undamped natural period of the accelerometer to the pulse duration of the excitation acceleration.

As indicated by Equation (2), when the pulse duration of the excitation acceleration is much larger than the undamped natural period of the accelerometer, namely R→0, the first two terms of the equation can be ignored and, therefore, ϕ≈α; when the pulse duration of the excitation acceleration decreases and R increases, the first two terms will be influenced. The first term will cause the oscillation of α and the second term will lead to a time lag.

Assume the input signal of the accelerometer as:

(3) $$\alpha (t)=\{\begin{array}{c} \sin(\frac{\pi }{\tau }t)\;0\leq t\leq \tau \\ 0\;t>\tau \end{array}$$

Then, the accelerometer’s response to the half-sine excitation signal can be expressed as:

(4) ϕ(t)=α(t)∗h(t)

When 0≤t≤τ, the response can be expressed as:

(5) $$\phi (t)=\sin\left(\frac{\pi }{\tau }t\right)*\left\{-\frac{\omega_{n}}{\sqrt{1-\zeta^{2}}}e^{-\zeta \omega_{n}t}\sin\left(\omega_{n}\sqrt{1-\zeta^{2}}t\right)\right\}$$

By solving Equation (5), we could obtain:

(6) $$\phi (t)=e^{-\zeta \omega_{n}t}\left\{c_{1}\cos\left(\omega_{n}\sqrt{1-\zeta^{2}}t\right)+c_{2}\sin\left(\omega_{n}\sqrt{1-\zeta^{2}}t\right)\right\}+\left\{a_{1}\cos\left(\frac{\pi }{\tau }t\right)+b_{1}\sin\left(\frac{\pi }{\tau }t\right)\right\}$$

where $a_{1}=\frac{A\omega_{n}^{2}d_{1}}{d_{1}^{2}+d_{2}^{2}}$, $b_{1}=-\frac{A\omega_{n}^{2}d_{2}}{d_{1}^{2}+d_{2}^{2}}$, $c_{1}=-\frac{A\omega_{n}^{2}d_{1}}{d_{1}^{2}+d_{2}^{2}}$, $c_{2}=-\frac{A\omega_{n}^{2}(\zeta \omega_{n}d_{1}-\frac{\pi }{\tau }d_{2})}{\omega_{n}\sqrt{1-\zeta^{2}}(d_{1}^{2}+d_{2}^{2})}$, $d_{1}=\frac{2\pi \zeta \omega_{n}}{\tau }$, $d_{2}=\omega_{n}^{2}-{\left(\frac{\pi }{\tau }\right)}^{2}$.

Under circumstances when damping is not considered, Equation (6) can be simplified as:

(7) $$\phi (t)=\left\{c_{1}\cos\left(\omega_{n}t\right)+c_{2}\sin\left(\omega_{n}t\right)\right\}+\left\{a_{1}\cos\left(\frac{\pi }{\tau }t\right)+b_{1}\sin\left(\frac{\pi }{\tau }t\right)\right\}$$

where $a_{1}=0$, $b_{1}=\frac{\omega_{n}^{2}}{\omega_{n}^{2}-{\left(\frac{\pi }{\tau }\right)}^{2}}$, $c_{1}=0$, $c_{2}=\frac{\omega_{n}\frac{\pi }{\tau }}{\omega_{n}^{2}-{\left(\frac{\pi }{\tau }\right)}^{2}}$.

By simplifying Equation (7), the following equation can be obtained:

(8) $$\phi (t)=\frac{1}{1-{\left(\frac{\pi }{\omega_{n}\tau }\right)}^{2}}\left(\sin\left(\pi \frac{t}{\tau }\right)-\frac{\pi }{\omega_{n}\tau }\sin\left(\omega_{n}(t-\tau )\right)\right)$$

Assume the response error as ε, and the system’s resonant oscillation superimposed on the response waveform amplitude is less than ε. Then:

(9) $$\left|\frac{\phi (t)}{a(t)}\right|\leq 1+\varepsilon$$

(10) $$\frac{\left|\left(\sin\left(\pi \frac{t}{\tau }\right)-\frac{\pi }{\omega_{n}\tau }\sin\left(\omega_{n}(t-\tau )\right)\right)\right|}{\left|1-{\left(\frac{\pi }{\omega_{n}\tau }\right)}^{2}\sin\left(\pi \frac{t}{\tau }\right)\right|}\leq 1+\varepsilon$$

As indicated by Equation (10):

(11) $$\frac{1+\frac{\pi }{\omega_{n}\tau }}{1-{\left(\frac{\pi }{\omega_{n}\tau }\right)}^{2}}\leq 1+\varepsilon$$

If $\omega_{n}=2\pi f_{x}$ and $f_{x}$ denotes the resonant frequency of the accelerometer, then:

(12) $$\tau \geq \frac{1+\varepsilon }{2\varepsilon f_{x}}$$

Equation (12) is the formula to solve the minimum width of the excitation pulse required by the quasi-static calibration of high-g accelerometers with different resonant frequencies. When τ, the pulse width of the excitation signal, is greater than the minimum width determined by Equation (12), the calibration error of the accelerometer is within the given range (Table 1).

## 3. The Quasi-Static Calibration Testing System

**Figure 5:**
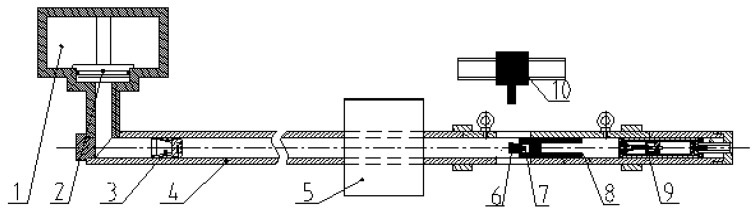
Schematic diagram of the quasi-static calibration system. (1) High-pressure gas chamber; (2) valve; (3) projectile; (4) launching tube; (5) silencer; (6) collision object (sensor mounting base); (7) calibrated accelerometer; (8) guiding cylinder; (9) liquid gas buffer device; and (10) laser interferometer.

At present, it is difficult for the commonly used high-impact excitation system to produce pulse signals with a pulse width over 300 μs, and it cannot be calibrated for high-g accelerometers with low resonant frequencies. Therefore, we develop a quasi-static calibration testing system for high-g accelerometers. As shown in Figure 5, a gas gun is used as the loading equipment to conduct quasi-static calibration of the high-g accelerometer assisted by a laser interferometer.

The calibration system in our lab is composed of an air gun, projectile, test component, and laser interferometer. The air gun is a one-stair air gun which is composed of a high pressure chamber with a volume of 0.036 m^3^, valve, silencer, liquid gas buffer device, launching canal with 6.2 m length, and 100 mm caliber. The collision surface of the projectile which can be filled with soft materials, such as felt, is made from steel, and other parts are made from aluminum alloy. The test component is composed of a collision object, calibrated accelerometer, and guiding cylinder. A diffraction grating is glued on the surface of the collision object.

The working principle of the whole calibration system is as follows: gas is injected into a high-pressure gas chamber and the valve is opened after the preset working pressure is reached. High-pressure gas is used to promote an accelerated movement of the projectile along the launching tube of the gas gun; driven by the high-pressure gas, the projectile collide with an object at a speed of 30–100 m/s. During collision, the collision object (installed with a high-g accelerometer) gains the accelerated speed and moves forward, along with the projectile, until it decelerates and stops under the influence of the liquid gas buffer device. To absorb the remaining energy after the collision, we applied technology similar to the recoil system for guns to construct a liquid gas buffer device which is composed of a large number of small holes using the resistance and energy consumption when the liquid passes through the small hole at a high speed. The laser interferometer measures the excitation acceleration signal through a diffraction grating attached to the surface of the collision object. After being converted by a charge or voltage amplifier, the output of the calibrated accelerometer is recorded in a transient recorder together with the signal output by the interferometer.

According to the method in ISO16063-13, the Doppler signals output by the laser interferometer can be used to determine the impact acceleration. As compared with the output of the calibrated accelerometer, its impact sensitivity can be determined by:

(13) $$S_{sh}=\frac{u_{peak}}{a_{peak}}$$

where $S_{sh}$ denotes the sensitivity of the high-g accelerometer; $u_{peak}$ denotes the peak output of the high-g accelerometer; and $a_{peak}$ is the peak value of the impact acceleration produced in the interferometer.

## 4. Factors Influencing Excitation Pulse Width

**Figure 6:**
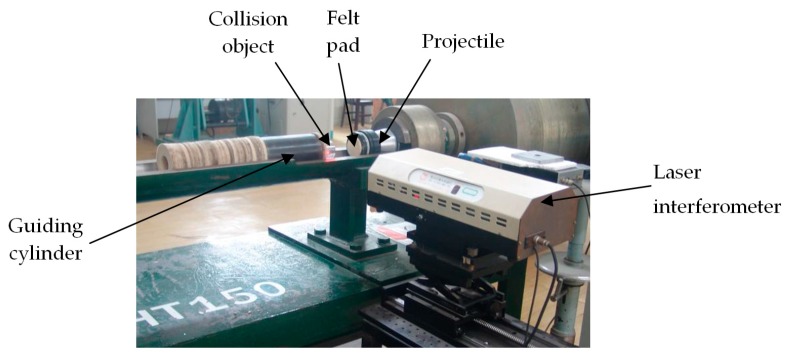
Photo of the projectile before collision with the collision object.

During calibration, the amplitude and pulse width of the acceleration signal obtained by the collision object can be adjusted by changing the pressure in the high-pressure gas chamber or changing the material and thickness of the collision surface [18]. During the test, the collision object produce an acceleration signal within amplitude of 10^5^ g through a frontal collision with the projectile, as shown in Figure 6. The felt pad is used as the collision surface, with a diameter of 50–100 mm, single-layer thickness of 10 mm, and a density of 0.255 g/cm^3^.

**Figure 7:**
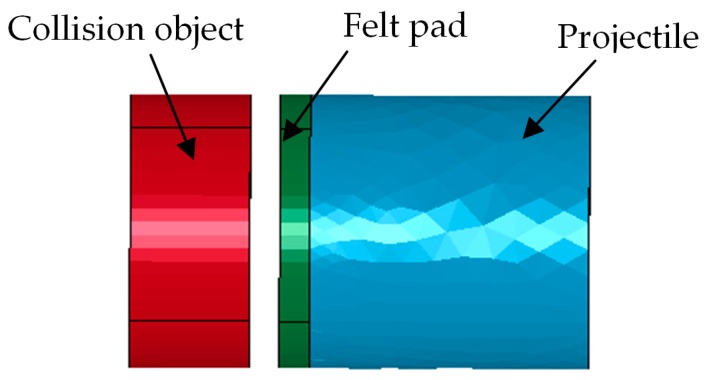
Finite element model of the collision process.

To obtain the excitation pulse width required by the accelerometer and reduce the number of required debugging tests, the dynamic process of the collision is simulated using ANSYS-LS-DYNA software (ANSYS Corporation, Canonsburg, PA, USA). Figure 7 shows the finite element model. A 3-D solid 164 unit is used, the impact velocity of the projectile is set as 50 m/s, and the terminal time is set as 1500 μs. The 3-D Lagrange rule is used to simulate the collision process by changing the mass of the collision object, the contact area, and the thickness of the felt pad.

### 4.1. Influence of the Collision Object’s Mass on Excitation Pulse

**Figure 8:**
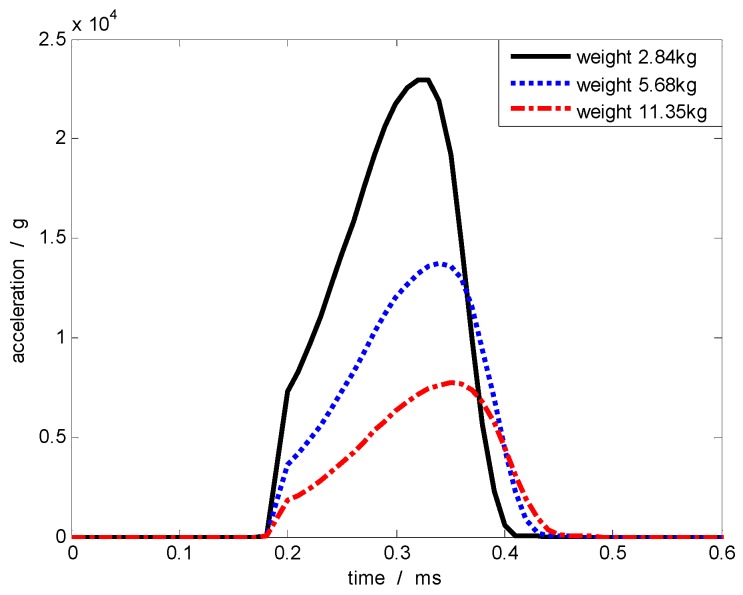
Acceleration pulse generated at the same projectile velocity but with different masses of the impacted body (diameter of the contact surface: 50 mm).

When the material of the collision object is certain, the kinetic energy lost during the collision is related to the mass ratio of the two objects. The kinetic energy lost is transformed into internal energy because of the permanent deformation generated on the objects. Therefore, when the mass of the gas gun projectile is constant (2.72 kg), the kinetic energy lost during the collision could be decreased by reducing the mass of the collision object. As indicated by Table 2, decreasing of the mass of the collision object has a relatively small impact on the pulse width, but has great influence on the acceleration amplitude. The corresponding acceleration curve is shown in Figure 8. Furthermore, when the projectile velocity remain constant, a high acceleration amplitude is obtained by reducing the mass of the collision object.

### 4.2. Influence of Collision Contact Area on Excitation Pulse

**Figure 9:**
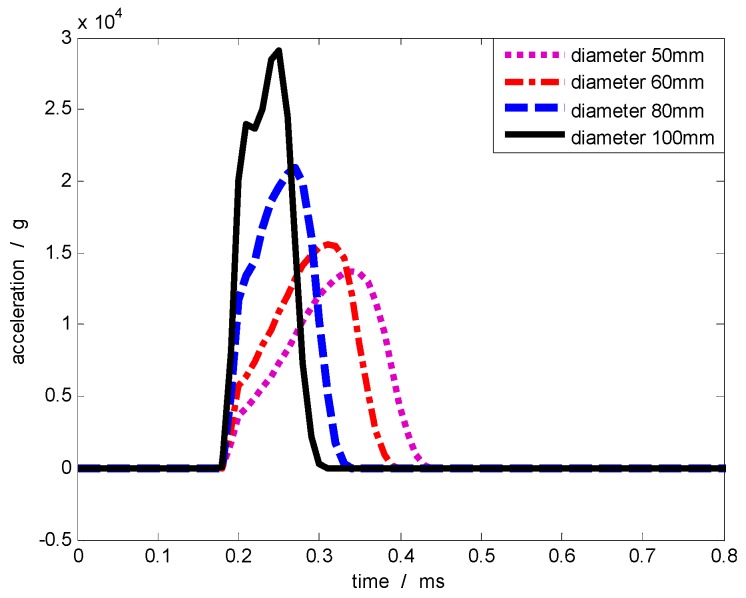
Acceleration pulse waveforms generated by different contact areas.

Table 2 also demonstrates that the contact area influences pulse width. When the contact area is small, the acceleration pulse is wide. Thus, when the projectile velocity remains constant (50 m/s) and the mass of the collision object is constant (5.68 kg), the collision simulation is conducted by changing the collision contact area as shown in Table 3. As the diameter of the contact surface decreases, the acceleration pulse width gradually increases. Figure 9 shows part of the acceleration pulse curves. Here, as the diameter of the contact surface decreases, the rising edge of the acceleration pulse become slower and the pulse width grew larger.

### 4.3. Influence of Felt Pad Thickness on Excitation Pulse

**Figure 10:**
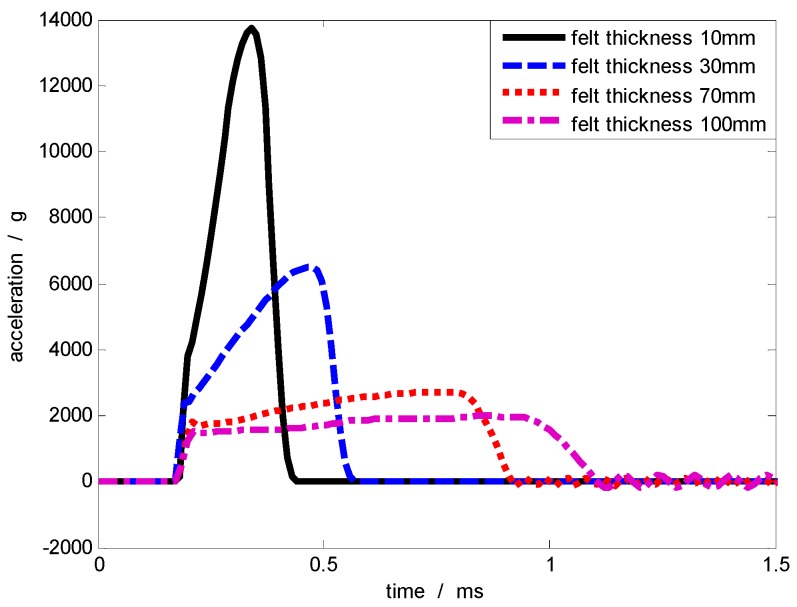
Acceleration pulse curves corresponding to different pad thicknesses.

With the diameter of the contact surface fixed at 50 mm, when the mass of the collision object remains constant (5.68 kg) and the projectile velocity is constant (50 m/s), the collision simulation is conducted by changing the thickness of the felt pad. Table 4 demonstrates the changes in the acceleration pulse when the thickness of the felt pad is within the range of 10–100 mm. As shown in Figure 10, as the thickness of the felt pad increases, the amplitude of the acceleration pulse declines, and the rising edge become slower, while the pulse width grew larger.

As indicated by the above analysis, this calibration system obtains acceleration signals with pulse widths greater than 300 μs by reducing the collision contact area and increasing the number of layers of the felt pad. Furthermore, high amplitude acceleration signals are gained by reducing the mass of the collision object and increasing the projectile velocity. To guarantee installation stiffness of the calibrated accelerometer and to prevent the collision object from being disturbed by vibrations of the launching tube during launch, the mass of the collision object cannot be too small; otherwise, the gathering of the laser on the raster surface will be affected, which will thereby result in failure of the test data.

## 5. Quasi-Static Calibration Test

A 988 piezoelectric high-g accelerometer from 204 institute has been used in this calibration system with a central compressed structure. Its range is within 10^5^ g with an inherent frequency of 125 kHz. Its first resonant frequency is near 40 kHz, as per the technical data. According to Equation (12), when the given calibration error is 3%, the minimum width of the excitation pulse should be 429 µs. During the calibration test, the mass of the collision object is 2.84 kg, the diameter of the collision contact surface is 50 mm, and the thickness of the felt pad is 50–70 mm. The excitation acceleration signals of different amplitudes generated by the projectile’s collision against the collision object at different speeds are adjusted by controlling the pressure inside the high-pressure gas chamber. The accelerometer is calibrated eight times within a quarter of the measuring range (Table 5 shows the detailed calibration data). The Doppler signals output by the laser interferometer and acceleration signals output by the calibrated accelerometer are recorded simultaneously in a transient waveform recorder. A Kistler Type 5011B is used as the charge amplifier (Winterthur, Switzerland).

**Figure 11:**
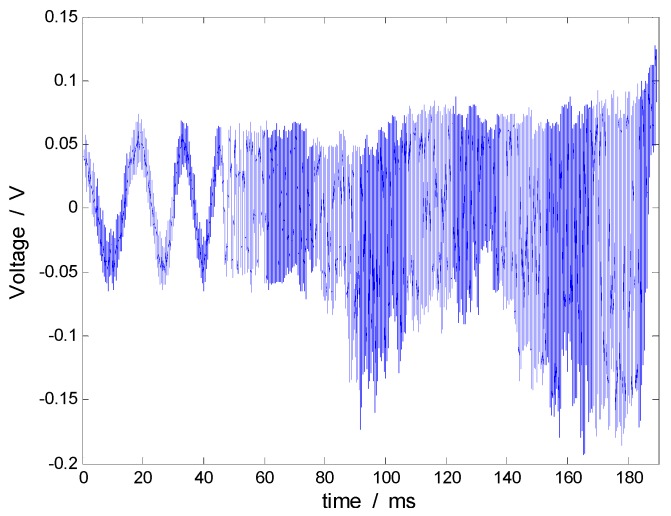
Doppler signal from the interferometer.

**Figure 12:**
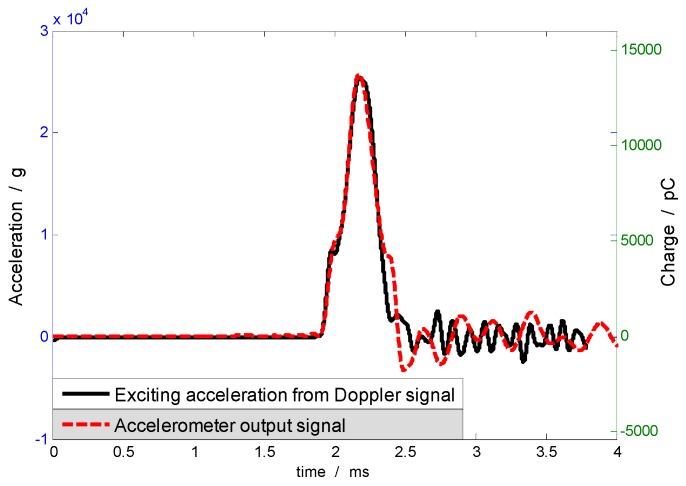
Excitation acceleration from laser interferometer and response acceleration from the accelerometer.

Figure 11 shows the Doppler signal by the laser interferometer and presents the test curve of the eighth calibration test in Table 5. The acceleration signal gained by Doppler signals output (Figure 11), namely, the excitation signal of the calibrated sensor, is shown by the solid line in Figure 12, and the peak acceleration $a_{peak}=$ 25,410 g. Meanwhile, the dotted line in Figure 12 demonstrates the signal output by the calibrated sensor, where the peak value $u_{peak}=$ 13,213 pC. According to Equation (13), the ratio of the peak values of these two signals is equal to the impact sensitivity in the table. The wide pulse calibration method avoids the filtering process of the response signal and improves the accuracy of peak sensitivity calibration.

**Figure 13:**
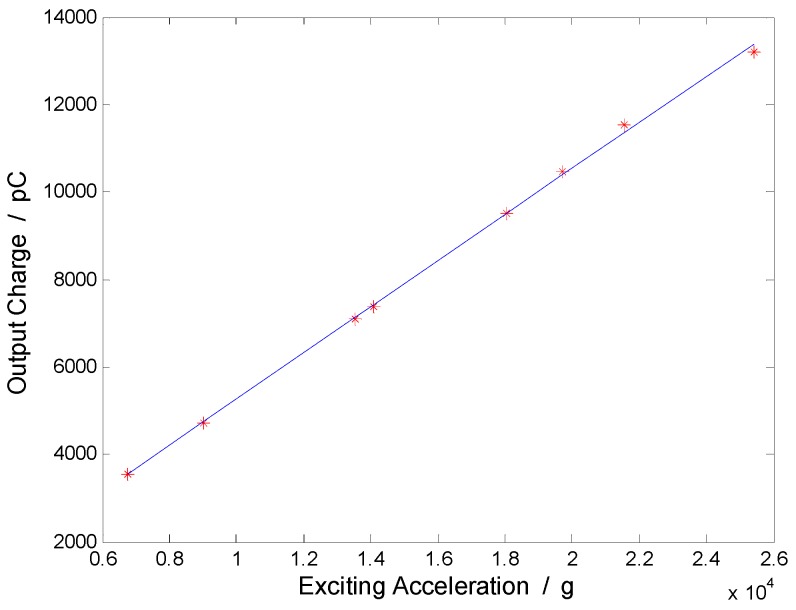
Fitted sensitivity curve of the calibrated accelerometer.

The calibration data in Table 5 are fitted by the least squares method, and the fitting curve is shown in Figure 13. If the fitting formula of the peak sensitivity is y=0.529x−36.490, then the response error of the calibrated accelerometer is shown in Table 5.

When the pulse width of the excitation signal is greater than the minimum width determined by Equation (12), the calibration error of the accelerometer is less than 3% (see Table 5).

As shown in Figure 12, the trend is nearly the same for response and excitation signals. The difference between two signals is due to the fact that the two signals come from different systems, resulting in different system characteristics. The measured signal from the laser interferometer is acceleration of the rigid body collision, without relation of the collision structure. Response acceleration of the collision object is the sum of the rigid body signal and the structure resonant signal. The pulse excitation signal with minimum width can only possibly reduce the response of the mount structure, but cannot be completely offset. 

## 6. Conclusions

By exploring the problem that impact sensitivity cannot be obtained accurately due to resonance during the quasi-static calibration of high-g accelerometers, this study contributed the following:

(1) A second-order mathematical model is used to describe the response characteristics of the high-g accelerometer, and deduces the relationship between and among the minimum pulse width of the excitation signals, resonant frequency, and calibration error of the calibrated accelerometer. With this method, the impact sensitivity of the calibrated accelerometer is gained within a certain error range. Increasing the width of the excitation pulse reduces amplitude changes of the response signal caused by resonant frequency, and effectively lowered the calibration error of impact sensitivity.
(2) The finite element method is applied to simulate the dynamic collision process. As indicated by our results, acceleration signals with a pulse width less than 1000 μs are obtained by changing the collision contact area and the number of layers of the felt pad; acceleration signals with an amplitude of 10^3^–10^5^ g are gained by changing the mass of the collision object and the projectile velocity.
(3) We conduct a quasi-static calibration test of a piezoelectric-type high-g accelerometer and apply the least squares method to obtain the impact sensitivity of the accelerometer from the calibration data. When the calibration error is 3%, the quasi-static calibration of high-g accelerometers with resonant frequency above 20 kHz is realized. Thus, high-g accelerometers calibrated using this method can satisfy the demands of engineering tests.

## Figures and Tables

**Table 1:** Minimum excitation pulse width of accelerometers with different resonant frequencies under undamped conditions.

Frequency $f_{x}(\mathrm{kHz})$		10	20	30	40	50	60
Pulse width τ (μs)	ε=1%	5050	2525	1683	1263	1010	842
	ε=3%	1717	858	572	429	343	286
	ε=5%	1050	525	350	263	210	175

**Table 2:** Influences of collision objects with different masses on excitation pulse width.

Collision Surface Diameter (mm)	Collision Object Mass (kg)	Acceleration Amplitude (g)	Acceleration Pulse Width (µs)	Speed (m/s)
100	11.35	15,859	140	12.19
	5.68	29,061	120	20.83
	2.84	49,087	110	32.21
50	11.35	7714	270	9.94
	5.68	13,730	260	17.22
	2.84	22,970	230	48.68

**Table 3:** Influences of different contact areas on excitation pulse width during collision.

Contact Surface Diameter (mm)	Acceleration Amplitude (g)	Acceleration Pulse Width (µs)	Speed (m/s)
50	13,730	260	17.22
60	15,480	220	19.60
70	18,280	190	19.86
80	20,950	160	19.50
90	24,500	140	20.00
100	29,061	120	20.83

**Table 4:** Influences of felt pad thickness on excitation pulse width.

Felt Pad Thickness (mm)	Acceleration Amplitude (g)	Acceleration Pulse Width (µs)	Speed (m/s)
10	13,730	260	17.22
30	6482	420	16.47
50	5832	725	15.43
70	2721	735	15.61
90	2249	865	15.20
100	1930	952	14.92

**Table 5:** Calibration data of a high-g accelerometer.

Test Number	Excitation Acceleration Peak Value (g)	Pulse Width (μs)	Output Charge Peak Value (pC)	Impact Sensitivity (pC/g)	Response Error ε (%)
1	6775	843	3529	0.521	2.07
2	9032	673	4724	0.523	1.53
3	13,550	599	7100	0.524	1.22
4	14,110	531	7379	0.523	1.40
5	18,060	523	9518	0.527	0.58
6	19,730	446	10,477	0.531	0.20
7	21,530	433	11,519	0.535	0.66
8	25,410	418	13,213	0.520	1.85

Notes: The pulse width in the above tables refers to the time duration from the excitation acceleration signal’s rise along 10% of the maximum peak to its decline along 10% of the maximum peak.
